# Dependence of cyanobacterium growth and Mars-specific photobioreactor mass on total pressure, pN_2_ and pCO_2_

**DOI:** 10.1038/s41526-024-00440-1

**Published:** 2024-11-02

**Authors:** Cyprien Verseux, Tiago P. Ramalho, Emma Bohuon, Nils Kunst, Viktoria Lang, Christiane Heinicke

**Affiliations:** 1 Center of Applied Space Technology and Microgravity (ZARM), University of Bremen, Bremen, Germany; 2 Center for Environmental Research and Sustainable Technology (UFT), University of Bremen, Bremen, Germany

**Keywords:** Microbiology, Biotechnology

## Abstract

In situ resource utilization systems based on cyanobacteria could support the sustainability of crewed missions to Mars. However, their resource-efficiency will depend on the extent to which gases from the Martian atmosphere must be processed to support cyanobacterial growth. The main purpose of the present work is to help assess this extent. We therefore start with investigating the impact of changes in atmospheric conditions on the photoautotrophic, diazotrophic growth of the cyanobacterium *Anabaena* sp. PCC 7938. We show that lowering atmospheric pressure from 1 bar down to 80 hPa, without changing the partial pressures of metabolizable gases, does not reduce growth rates. We also provide equations, analogous to Monod’s, that describe the dependence of growth rates on the partial pressures of CO_2_ and N_2_. We then outline the relationships between atmospheric pressure and composition, the minimal mass of a photobioreactor’s outer walls (which is dependent on the inner-outer pressure difference), and growth rates. Relying on these relationships, we demonstrate that the structural mass of a photobioreactor can be decreased – without affecting cyanobacterial productivity – by reducing the inner gas pressure. We argue, however, that this reduction would be small next to the equivalent system mass of the cultivation system. A greater impact on resource-efficiency could come from the selection of atmospheric conditions which minimize gas processing requirements while adequately supporting cyanobacterial growth. The data and equations we provide can help identify these conditions.

## Introduction

Bioproduction systems that rely on diazotrophic, rock-weathering cyanobacteria for in situ resource utilization (ISRU) have been proposed as means of increasing the sustainability of crewed missions to Mars^1^. According to this proposal, cyanobacteria, samples of which would be stored in a refrigerated or desiccated state until inoculation, would be fed with water mined from the ground; carbon and nitrogen sourced from the atmosphere^2^; and the local regolith, from which it has been argued that they could extract the other necessary nutrients^3,4^. They would produce various consumables directly, such as dioxygen and dietary supplements, but also support the growth of secondary producers (e.g., plants^5^ or microorganisms^2,6,7^). These secondary producers could, in turn, generate a wide range of consumables including food, medicines, fuel precursors and structural materials^8,9^.

We previously performed proofs-of-concept that suggest the feasibility of feeding selected cyanobacteria solely with Martian resources^2^. However, whether the resource-efficiency of cyanobacterium-based production warrants its integration into Mars mission architectures remains unknown. The determination of this resource-efficiency is hindered by a lack of knowledge on cyanobacterium responses to the factors which are expected to limit their productivity on Mars. One of these factors pertains to the suitability of Mars’s regolith as feedstock, which is the object of a separate line of work^4^. Another is the behaviour of cyanobacteria under atmospheric conditions that differ from Earth-ambient. Indeed, cyanobacteria would not be cultivated in crewed compartments but in cultivation modules which feature different atmospheric conditions. It has been proposed that for highest resource-efficiency, these conditions would approach those of Mars^2,10^. First, relying on a gas composition closer to Mars-ambient would limit the need for gas processing and for importing consumables. Second, decreasing the pressure would relax constraints on the container’s structure (leading to a lower imported mass through, e.g., thinner walls), reduce the energy demand for pressurization, and help lower the rates of gas leakage. However, atmospheric conditions for cultivation cannot be exactly Mars-ambient: the total pressure (6.1 hPa on average at altitude zero, with variations based on altitude, season and time of day) is too low to sustain the metabolism of most microorganisms^11,12^, or to maintain liquid water at temperatures adequate for cyanobacterium cultivation. Besides, the partial pressures of metabolizable gases – CO_2_ and N_2_ – are not optimal. That of CO_2_ (pCO_2_) is higher than on Earth and may be suitable: 5.8 hPa under a total pressure of 6.1 hPa, assuming 95% CO_2_^13^, in contrast to a current value of 0.4 hPa on Earth at sea level. Conversely, the fraction represented by N_2_ is very low: circa 2.8%^13^, resulting in a partial pressure of N_2_ (pN_2_) below 0.5 hPa at ground level, which – as elaborated below – would hardly support a diazotrophic metabolism, if at all. The atmospheric conditions on the Martian surface would therefore not allow the growth of cyanobacteria.

A gas flow from the Martian atmosphere could be processed toward a higher pressure and nitrogen fraction. This may not need to be performed, or performed entirely, for the sole purpose of cyanobacterium cultivation: side products from CO_2_ purification systems, considered for other applications on Mars (e.g., fuel production), may for instance have a pressure higher than Mars-ambient and be enriched in nitrogen. Whether or not that is the case, processing samples of Mars’s atmosphere (or further processing waste gas from other systems) will be resource intensive. Optimizing resource-efficiency will therefore require a compromise between minimizing gas processing and maximizing biological productivity. As a proof-of-concept, diazotrophic cyanobacteria were previously cultivated under a low pressure (100 hPa) of gases available on Mars: 96% N_2_ and 4% CO_2_ (not accounting for the partial pressure of water vapour)^2^. Growth was vigorous but atmospheric conditions were not optimized for resource-efficiency. Optimization will require a fine knowledge of the relationship between cyanobacterium productivity and total pressure, pCO_2_ and pN_2_.

The minimum total pressure a microorganism can withstand without growth impairment is species-dependent^14^. Only a limited number of microbiology experiments have been performed at low but growth-permissive pressure, and only a small fraction of them have involved photosynthetic microorganisms. Some featured (non-cyanobacterium) microalgae of the genus *Chlorella*. They showed no negative impact of lowering the pressure to 565 hPa^15^, and even suggested a positive impact of decreasing it to 250 hPa^16^ under constant pCO_2_. Other low-pressure experiments included cyanobacteria. Unfortunately, none singled out the effects of the gases’ partial pressures. The growth of the cyanobacteria *Microcystis aeruginosa, Merismopedia* sp., and *Anabaena* spp. in nitrate-rich medium was shown to be lower under 0.5 bar of ambient air than under 1 bar^17^, but the atmospheric composition was constant, leading to, notably, a two-fold decrease in pCO_2_ – which was presumably limiting at one bar of ambient air already. Similarly, the growth of various photosynthetic microorganisms was monitored at low pressure (670, 330 and, for some, 160 and 80 hPa)^18^, but under a CO_2_ atmosphere (with fixed nitrogen provided in the medium), which does not enable one to discriminate between the effects of the reduced pCO_2_ and those of the reduced total pressure. The cyanobacterium among the tested strains, *Spirulina platensis*, failed to grow under 670 hPa (no results were provided for other pressure values), possibly due to the toxicity of the high pCO_2_. More conclusive insights were brought by experiments where the cyanobacteria *Synechocystis* sp., *Arthrospira platensis*, and *Anabaena cylindrica* grew at least as efficiently under 100 hPa as under 1 bar^19,20^. While joint effects of the variations in pCO_2_ and in total pressure cannot be ruled out entirely, pCO_2_ was presumably non-limiting and (for part of the samples) non-toxic, and the results strongly suggest that the impact on productivity of decreasing pressure down to 100 hPa is low at most. This is consistent with the body of work performed with other bacteria: most seem unaffected by low pressure per se down to this threshold^12^.

Constraints on the total pressure in an ISRU-based cultivation system, however, are not posed only by the effects of pressure in itself: assuming ideal gas behaviour, the total gas pressure is equal to the sum of the partial pressures of the individual gases (Dalton’s law), including pCO_2_ and pN_2_. pCO_2_ need not be high: Murukesan et al. (2015), for instance, found that pCO_2_ was non-limiting in their cyanobacterium cultivation setup from circa 4 hPa^20^. When relying on N_2_ as a nitrogen source, and under conditions that optimize productivity, the contribution of pN_2_ to the total pressure would however be more substantial. Reducing pN_2_ down to below 500 hPa was shown to limit the growth of *A. cylindrica* and *A. variabilis*, though growth was still vigorous at 100 hPa^21^. Those results are consistent with studies on non-cyanobacterial diazotrophs: the growth rates of *Azotobacter vinelandii* and *Azomonas agilis* were found to decrease with pN_2_ down from about 400 hPa^22^, and nitrogen fixation in *Beijerinckia indica* and *B. lacticogenes* was shown to increase with pN_2_ between 5 and 400 hPa. While none of the studies mentioned above systematically correlated cyanobacterial growth rates with pN_2_, they do suggest that reaching a pN_2_ high enough for vigorous growth by simply compressing the Martian atmosphere would require pressures several times that of the Earth at sea level.

Here we aim to provide knowledge required to optimize the atmospheric conditions in a photobioreactor that relies on Mars ISRU. We determine how the growth rates of *Anabaena* sp. PCC 7938 – a strain previously selected as a model for cyanobacterium-based ISRU on Mars^5^ – depend on pCO_2_ and pN_2_, as well as on low pressure in itself (i.e., excluding the effects of the partial pressures of metabolizable gases) down to 80 hPa. We use our results to estimate the minimum pressure required to sustain given growth rates, or given productivities, of cyanobacteria. We then assess the impact of pressure on the minimal mass of an airlift photobioreactor’s outer walls, considering that these walls must withstand the inner-outer pressure difference. The experimental data and sets of equations we present provide a basis for identifying, in any Mars mission scenario, the right compromise between minimizing gas processing and maximizing cyanobacterial productivity.

## Methods

### Model organism and routine growth conditions

*Anabaena* sp. PCC 7938 (hereafter, *Anabaena* sp.) was obtained from the Pasteur Culture Collection of Cyanobacteria (Paris, France). Prior to experiments, cultures were grown inside a poly klima PK 520-LED photo-incubator at 25 °C, in BG11_0_ medium, with a light intensity of 15–20 μmol_ph_ m^−2^ s^−1^ (16 h/8 h day/night cycle), on a rotary shaker set at 100 rpm.

### Setup for cultivation under artificial atmospheres

**Fig. 1:**
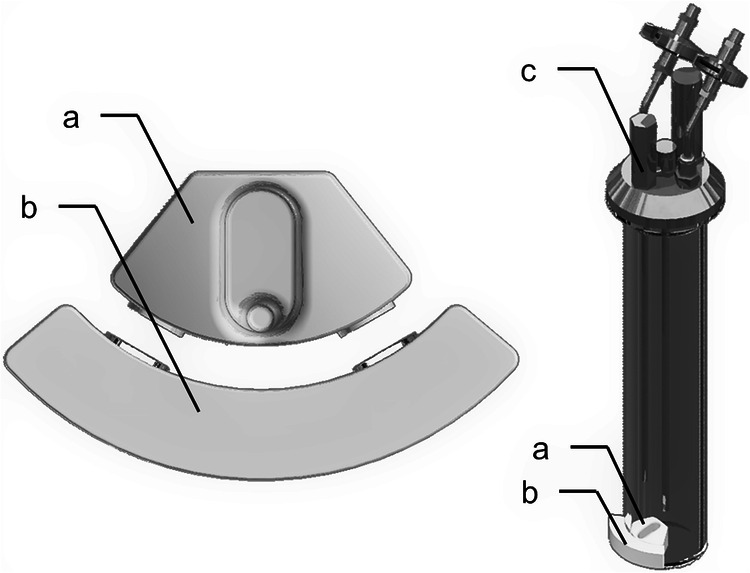
System integrated to Atmos for sampling cultures without altering the atmospheric conditions in the vessels. Left: Close-up of the sample lifting system, composed of a Teflon sample container (**a**) placed inside the vessel and of an outer piece (**b**) connected to it by two pairs of neodymium magnets. Right: View of one vessel. Liquid samples can be brought to within a few centimetres of the lid by using the sample lifting system, then collected with a syringe and a needle inserted through a sampling port (**c**) integrated into the lid.

During experiments, *Anabaena* sp. was grown inside Atmos, a photobioreactor developed in-house for the cultivation of phototrophic microorganisms under low pressure^2^. This hardware was modified to integrate a system (shown in Fig. 1 and outlined hereafter) which enables taking samples without affecting the atmospheric conditions inside the vessels. An injector nut with support and ¼” low bleed septum (Vici Valco Instruments, Houston, TX) was fitted, with a straight male connector (Swagelock Company, Solon, OH), to one of the G1/4” holes in the lid of each vessel. Prior to liquid sampling, the culture was stirred vigorously, and 1.3 ml was lifted to within centimetres of the lid, by using a Teflon sample container placed inside the vessel and manually controlled with an outer piece (3D-printed in-house). This container was designed in-house and manufactured by Spanflug Technologies GmbH (Munich, Germany). One millilitre of culture was then sampled with a syringe (Model 1002; Hamilton Company, Reno, NV) and a needle (23 gauge, 100 mm, removable; Hamilton Company) inserted through the septum. To reduce the risks of inward biological contamination, the needle and the top of the injector nut were sterilized prior to sampling by, respectively, passing through a flame or wiping with 70% ethanol. During this sterilization step and until the needle had been fully inserted, the flame of a Bunsen burner was maintained above the lid.

### Cultivation under various partial pressures of carbon dioxide and dinitrogen

Atmos was used to assess the relationship between the growth rates of *Anabaena* sp. and the partial pressures of CO_2_ and N_2_. In each vessel, 70 ml of BG11_0_ was inoculated to an optical density at 750 nm (OD_750_) of 0.05 with precultures in late exponential phase (one preculture was used for all vessels within one experimental run). When the partial pressure of CO_2_ was varied across samples, the medium was buffered with 20 mM HEPES to avoid large changes in pH.

At the onset of experiments, the air in the vessels was replaced with a gas mixture – provided by either Linde Gas (Dublin, Ireland; standard gas mixture class 1) or Air Liquide S.A. (Paris, France; CYSTAL mixture) – containing either 1% CO_2_ and one among various fractions of N_2_ (0, 2, 10, 20 or 80%), or 80% N_2_ and one among various fractions of CO_2_ (0, 0.05, 0.5, 1, 2 or 5%), and Ar as a balance gas. Six replicate vessels (across at least two separate experimental runs) were used per gas mixture. The pressure in the gas phase was set to 1000 hPa and automatically reset to this value when deviations exceeded 10 hPa. The gas in the headspace was renewed (by flushing at constant pressure) 2 h after starting the experiment, then every 12 h throughout the experiment. The light intensity was set to 50 μmol photons m^−2^ s^−1^ per side (measured at the inner side of the vessel, where it is closest to the LED strips, at culture mid-height), temperature to 30 °C and stirring to 100 rpm.

*Anabaena* sp. was grown in these conditions for four days. Every 24 h, samples were taken to measure the OD_750_.

### Cultivation under various total pressures

Cultivation assays were used to test whether relying on a low total pressure (down to 80 hPa) has effects which are independent of pN_2_ and pCO_2_ on *Anabaena* sp.’s growth rates. They were performed as described above but for the following modifications. *Anabaena* sp. was cultivated in 80 ml of BG11 (BG11_0_ supplemented with 1.5 g l^−1^ NaNO_3_) buffered with 20 mM HEPES and supplemented with 10 g l^−1^ NaHCO_3_. The air in the culture vessels was evacuated down to 1000, 100 or 80 hPa and replaced with Ar (Argon 6.0, Linde Gas). The gas in the headspace was renewed 2 h after starting the experiment, then every 24 h throughout the experiment. The pressure was automatically reset to its target value when it deviated by more than 10 or (for vessels under 80 hPa) 5 hPa.

### Assessment of growth rates as a function of gas composition and pressure, assuming liquid-gas equilibrium

As no total pressure-dependent effect was found over the tested range (80–1000 hPa), the growth rates of *Anabaena* sp. were determined as a function of the partial pressures of CO_2_ and N_2_ (over 0–50 hPa and 0–800 hPa, respectively). Growth rates were determined based on OD_750_ values in exponential phase and fitted to a Monod equation using the non-linear curve fitting function of Prism (GraphPad Software, Boston, MA; version 10.2.0 for Windows). As CO_2_ appeared to be limiting in some of the experimental conditions aimed at assessing the effect of pN_2_, a Monod equation for pN_2_ in the absence of CO_2_ limitation was obtained by excluding these conditions and including the 2%-CO_2_, 80%-N_2_ condition (R^2^ = 0.995). Growth rates under given sets of atmospheric conditions were then determined as the minima between the growth rates predicted based on pN_2_ and these predicted based on pCO_2_.

### Assessment of the minimum gas pressure required to sustain given growth rates, assuming liquid-gas equilibrium

The minimum gas pressure (*P*_*ga*s_, hPa) required to sustain given specific growth rates of *Anabaena* sp. was determined as the sum of pCO_2_, pN_2_, and the partial pressures of water vapour (pH_2_O) and of gases injected alongside nitrogen (pOthers):

1 $${P}_{{gas}}={{\rm{pCO}}}_{2}+{{\rm{pN}}}_{2}+{{\rm{pH}}}_{2}{\rm{O}}+{\rm{pOthers}}$$

The O_2_ produced by cyanobacteria was neglected as we assume a constant gas flow which prevents its accumulation. pCO_2_ and pN_2_ were calculated based on equations analogous to Monod’s (see above). pOthers accounts for gases present in the source of N_2_, which we assume to be derived from pressurized Martian atmosphere by removal of CO_2_ but not of other gases. We also assume that the ambient (unprocessed) atmosphere has the following composition^13^: 94.9% CO_2_, 2.79% N_2_, 2.08% Ar, 0.174% O_2_, and 0.075% CO. Assuming ideal gas behaviour (a reasonable approximation given the low pressure range and the temperature around 30 °C), it follows that:

2 $${\rm{pOthers}}={{\rm{pN}}}_{2}\cdot \frac{2.33}{5.12}$$

pH_2_O was determined as follows. The water vapour pressure that would be saturating if no other gases were present (${p}_{H2O,{pure},s}$, hPa) was first calculated as a function of the system’s temperature^23^ (*T*, K):

3 $$\begin{array}{l}{p}_{H2O,{pure},s}=\\{0.01\,\cdot\, e}^{-6024.5282\,\cdot\, {T}^{-1}+21.2409642-2.711193\,\cdot\, {10}^{-2}\,\cdot\, T+1.673952\,\cdot\, {10}^{-5}\,\cdot\, {T}^{2}+2.433502\,\cdot\, \mathrm{ln}T}\end{array}$$

The water vapour pressure in the absence of other gases (${p}_{H2O,{pure}}$, hPa) is dependent on relative humidity (*%RH*, %):

4 $${p}_{H2O,{pure}}=\frac{{p}_{H2O,{pure},s}\,\cdot\,\% {RH}}{100}$$

Here we assume a relative humidity of 100%, hence ${p}_{H2O,{pure}}={p}_{H2O,{pure},s}$.

${p}_{H2O,{pure}}$ pertains to a system where water vapour is the only gas. To account for the impact of other gases, a dimensionless enhancement factor, *f*_*w*_, was applied:

5 $${p}_{H2O}={p}_{H2O,{pure}}\,\cdot\, {f}_{w}$$

This factor was calculated as follows^24^ (where *P*_*Earth*_ [hPa] is the ambient pressure on Earth at sea level):

6 $$\begin{array}{l}{f}_{w}\left(P,{t}_{{dp}}\right)=1+\frac{{10}^{-6}\cdot {p}_{H2O,{pure},s}\cdot 100}{{t}_{{dp}}}\cdot \left[\left(38+173\cdot {e}^{-\frac{{t}_{{dp}}}{43}}\right)\cdot \left(1-\frac{{p}_{H2O,{pure},s}}{{P}_{{Earth}}}\right)\right.\\\left.\qquad\qquad\,+\left(6.39+4.28\cdot {e}^{-\frac{{t}_{{dp}}}{107}}\right)\cdot \left(\frac{{P}_{{Earth}}}{{p}_{H2O,{pure},s}}-1\right)\right]\end{array}$$

The constants in Eq. (6) were determined by others, empirically, for a terrestrial atmospheric composition. Their use in the present work was deemed acceptable given the similar elemental composition of the expected gas phase (an N_2_-dominated mixture of the same main components, though at different concentrations) and the low relative impact (<10% increase) of the enhancement factor on the calculated vapour pressure. ${t}_{{dp}}$ (K) is the dew point temperature, which we determined with an empirical equation^25^:

7 $${t}_{{dp}}=\frac{243.12\cdot {ln}\left(\frac{{p}_{H2O,{pure},s}}{611.2}\right)}{17.62-{ln}\left(\frac{{p}_{H2O,{pure},s}}{611.2}\right)}$$

### Assessment of the minimum inner pressure under constrained superficial velocity of sparged gases

The partial pressures calculated as described above assume an equilibrium between the liquid and gas phases, and that the dissolved inorganic carbon and nitrogen are replenished as they are consumed by cyanobacteria. Maintaining these conditions requires gases to be sparged with high enough a superficial velocity. However, superficial velocity can be increased to only some extent before inducing excessive shear stress (it is common practice not to exceed 0.08 m s^−1^). Keeping growth rates constant as productivity goes up may therefore require enhancing the input of dissolved gases by increasing pCO_2_ and pN_2_. This effect – as well as that of the medium’s hydrostatic pressure – on the minimum inner pressure of a photobioreactor was accounted for as described below.

Hereafter, the concentrations of dissolved inorganic carbon and dissolved CO_2_ are used interchangeably. While this is not formally correct as the former is also composed of carbonate and bicarbonate, inorganic carbon is entirely replenished by CO_2_, and the half-velocity constant determined as described above already accounts for it.

Assuming ideal gas behaviour, the required partial pressure of gas *i* (*p*_*i*_, hPa) – *i* standing for either CO_2_ or N_2_ – can be expressed as a function of its molar concentration in the gas phase (${C}_{i,G}$, mol_*i*_ m^–3^):

8 $${p}_{i}=\left(\frac{{C}_{i,G}}{100}\right)\cdot R\cdot T$$

${C}_{{i,G}}$ is dependent on the gas transfer rates (${iTR}$, mol_*i*,*L*_ m^–3^ s^–1^) and gas-liquid mass transfer coefficients (${k}_{{iL}}a$, s^–1^) of CO_2_ and N_2_. This relationship is shown in Eq. (9), where *m*_*i*_ (mol_*i*__*,G*_ mol_*i,L*_^–1^) is the partition coefficient – the ratio of the concentrations of gas *i* in the liquid and gas phases at equilibrium – calculated based on Henry’s law, and ${C}_{i,L}$ (mol_*i,L*_ m^–3^) is the concentration of CO_2_ or N_2_ in the medium.

9 $${C}_{i,G}={m}_{i}\cdot \left(\frac{{iTR}}{{k}_{{iL}}a}+{C}_{i,L}\right)$$

The *iTR* required to replenish the dissolved CO_2_ and N_2_ was assessed based on the mass balance, shown in Eq. (10), over the liquid phase. The concentration of each dissolved gas is determined by the following elements: the ingoing and outgoing liquid flows (${F}_{L,{in}}\,{\rm{and}}\,{F}_{L,{out}}$, m^3^ s^–1^) per culture volume (${V}_{{culture}}$, m^3^); the gas concentrations in the ingoing and outgoing liquids (${{C}_{i,L,{in}}\,{\rm{and}}\,{C}}_{i,L,{out}}$, mol_*i*_ m^–3^); the gas-liquid mass transfer (${iTR}\cdot {V}_{{culture}}$, mol_*i*__*,L*_ s^–1^); and the gas consumption rate by cyanobacteria. The latter was calculated as the product of biomassproductivity ($\frac{{{dC}}_{x}}{{dt}}$, mol_x_ m^–3^ s^–1^) and molar coefficient per mol of carbon in the biomass – i.e., the number of moles of gas *i* consumed for producing an amount of biomass that contains one mole of carbon (${i}_{{\rm{COEFF}}}$, mol_*i*_ mol_x_^–1^; here we assume^26^ values of 1 for CO_2_ and 0.0873 for N_2_).

10 $$\frac{d{C}_{i,L}}{{dt}}=0=\frac{{F}_{L,{in}}}{{V}_{{culture}}}\cdot {C}_{i,L,{in}}+\frac{{F}_{L,{out}}}{{V}_{{culture}}}\cdot {C}_{i,L,{out}}+{iTR}-{i}_{{\rm{COEFF}}}\cdot \frac{{{dC}}_{x}}{{dt}}$$

The concentrations of dissolved gases are assumed to remain constant, hence $\frac{{dCi},{L}}{{dt}}=0$. Assuming batch cultivation (and therefore, that ${{F}_{L,{in}}}={{F}_{L,{out}}}=0$), ${{iTR}}={{{i}_{\rm{COEFF}}}}\cdot \frac{{dC}_{x}}{dt}$.

${k}_{{iL}}a$ (used in Eq. (9)) can be calculated from the gas-liquid mass transfer coefficient of O_2_ (${k}_{O2L}a$, s^−1^) through Eq. (11), where *D*_*i*_ (cm^2^ s^−1^) is the diffusion coefficient of gas *i* and *D*_*O*2_ (cm^2^ s^−1^) that of O_2_ (here we assume *D*_*O*2_ = 2.42 ×10^−5^, *D*_*CO2*_ = 1.91 ×10^−5^, and *D*_*N2*_ = 2.00 ×10^−5^):

11 $${k}_{{iL}}a={k}_{O2L}a\cdot \sqrt{\frac{{D}_{i}}{{D}_{O2}}}$$

While diffusivity coefficients vary with pressure, at a given temperature, their ratio does not. ${k}_{O2L}a$ was calculated using Eq. (12):

12 $${k}_{O2L}a={\sqrt{\frac{{P}_{{Earth}}}{{P}_{{gasR}}}}}\,\cdot\,{{g}\,_{{corr}}}\,\cdot\,0.32\cdot {\left({v}_{{gs}}\cdot \frac{{P}_{{gas}}}{{P}_{{gasR}}}\right)}^{0.7}$$

In Eq. (12), a factor is applied to the superficial velocity of the incoming gas (*v*_*gs*_, here assumed to be 0.08 m s^−1^) to account for a variation in velocity along the height of the liquid phase. ${P}_{{gasR}}$ (hPa) is the gas pressure at culture mid-height. As shown in Eq. (13), its value depends on hydrostatic pressure (${P}_{{hyd}}$, hPa), which itself depends on Martian gravity (*g*_*Mars*_, equal to 3.7278 m s^–2^), medium density (ρ_*L*_, here assumed to be 1000 kg m^−3^) and culture height (${h}_{L}$, m).

13 $${P}_{{gasR}}={P}_{{gas}}+0.5\,\cdot\,{P}_{{hyd}}={P}_{{gas}}+0.5\,\cdot\,\frac{{g}_{{Mars}}\,\cdot\,{{{\rho }}}_{L}\,\cdot\,{h}_{L}}{100}$$

The 0.32 factor and 0.7 exponent in Eq. (12) are empirical constants for a bubble column with normal bubble aeration^27^. Equation (12) also includes the following two correction factors. One ($\sqrt{\frac{{P}_{{Earth}}}{{P}_{{gasR}}}}$) accounts for an increase in gas-liquid mass transfer with decreasing pressure, which is due to an increase in gas diffusivity. This correction was defined by considering that, in all of the various models which have been proposed to assess ${k}_{L}a$, the latter varies proportionally to the square root of the diffusion coefficient^28^, which itself is inversely proportional to pressure^29^. It is only applicable because the empirical constants pertain to a photobioreactor under Earth’s ambient pressure at sea level. It is worth noting that the effects of diffusivity on gas-liquid mass transfer coefficients are usually not considered when modelling bioprocesses. This is mostly because they are not as significant as the associated change in bubble volume. As the bubble volume is here assumed to be constant, these effects are more relevant. The second correction factor addresses the lower gas-transfer efficiency in low gravity environments (${g}_{{corr}}$), calculated as described by others^30^. It accounts for the decrease in bubble velocity, which results in lower turbulent mixing, and is only valid when the bubble volume remains constant across the compared gravity levels.

### Assessment of a photobioreactor’s wall mass as a function of total inner pressure

Increasing the difference between the inner and outer pressures increases the stress on the photobioreactor’s walls, and thereby the minimum thickness required of these walls to withstand it. To illustrate the impact this would have on payload mass, rough calculations were performed based on a simple structure which approximates that of a bubble column photobioreactor: a cylinder closed by plates at both ends, with a liquid phase of one cubic metre and whose diameter is twice its height, with a headspace increasing the height by one tenth, and under a constant temperature of 30 °C.

The shear forces acting on the cylinder are dependent on the inside-outside pressure difference ($\Delta {P}_{{cyl}}$, hPa). The inner pressure is the sum of the inner gas pressure (${P}_{{gas}}$) and the hydrostatic pressure (${P}_{{hyd}}$), and the outer pressure is that of the ambient atmosphere (*P*_*Mars*_, here assumed to be 6 hPa):

14 $${\Delta P}_{{cyl}}={P}_{{gas}}+{P}_{{hyd}}-{P}_{{Mars}}$$

The forces acting on the on the upper plate – the lid – are dependent on the difference between ${P}_{{gas}}$ and ${P}_{{Mars}}$ ($\Delta {P}_{{top}}$, hPa).

15 $$\Delta {P}_{{top}}={P}_{{gas}}-{P}_{{Mars}}$$

The minimum t hickness of the cylindrical wall necessary to withstand ${\Delta P}_{{cyl}}$ (${s}_{{cyl},\min }$, m) was calculated for different materials – poly(methyl methacrylate) (PMMA), polyethylene terephthalate (PET), polycarbonate (PC), aluminium (alu) and steel – using Eq. (16) (based on the maximum shear stress theory)^31^:

16 $${s}_{{cyl,min}}=\frac{\Delta {P}_{{cyl}}\cdot d}{2\cdot \frac{{R}_{m}}{S}-{\Delta P}_{{cyl}}}$$

In Eq. (16), *d* (m) is the cylinder’s inner diameter, *R*_*m*_ is the tensile strength of material *m* (hPa; here we assume^32,33^ *R*_*PMMA*_ = 73 ×10^4^; *R*_*PET*_ = 80 ×10^4^; =; *R*_*PC*_ = 66 ×10^4^; *R*_*Alu*_ = 250 ×10^4^; and *R*_*Steel*_ = 650 ×10^4^) and S is a safety factor (here set to 2).

A lower boundary on material thickness (*s*_*min,m*_, m; here assumed to be 2 ×10^−3^ for steel and 3 ×10^−3^ for the other materials^32,33^) was set to account for constraints on manufacturing and handling. The thickness of the cylindrical wall (*s*_*cyl*_, m) is therefore assumed to be the minimum between *s*_*cyl,min*_ and *s*_*min,m*_.

Based on $\varDelta {P}_{{top}}$ (Eq. (15)), the minimum thickness of the lid (*s*_*top,min*_, m) was calculated according to Eqs. (17)–(20)^34,35^:

17 $${s}_{{top},{min}}={C}_{1}\cdot \left(d-2\,\cdot\,{s}_{{cyl}}\right)\cdot \sqrt{\frac{\varDelta {P}_{{top}}\cdot S}{{R}_{m}}}$$

18 $${C}_{1}={max} \left(0.40825\cdot {A}_{1}\cdot \frac{d+{s}_{{cyl}}}{d}{;}\,0.299\cdot \left(1+1.7\cdot \frac{{s}_{{cyl}}}{d}\right)\right)$$

19 $${A}_{1}={B}_{1}\cdot \left(1-{B}_{1}\cdot \frac{{s}_{{cyl}}}{2\cdot \left(d+{s}_{{cyl}}\right)}\right)$$

20 $$\begin{array}{c}{B}_{1}=1-\frac{3{\cdot R}_{m}}{{\varDelta P}_{{top}}\cdot S}{\cdot \left(\frac{{s}_{{cyl}}}{d}\right)}^{2}+\frac{3}{16}{\cdot \left(\frac{{s}_{{cyl}}}{d}\right)}^{4}\cdot \frac{{\varDelta P}_{{top}}\cdot S}{{R}_{m}}-\frac{3}{4}\\ \cdot \frac{\left(2\cdot d+{s}_{{cyl}}\right)\cdot {s}_{{cyl}}^{2}}{{\left(d+{s}_{{cyl}}\right)}^{3}}\end{array}$$

As the bottom plate is assumed to be resting on a surface (and thus not to be directly impacted by the inner-outer pressure difference), its thickness is assumed to be equal to ${s}_{\min ,m}$.

The mass of the photobioreactor’s walls was determined as the sum of the masses of the cylinder, lid and bottom plate. These individual masses were obtained by multiplying the volume (m^3^) of each component by the density of the material it is made of (ρ_*m*_, kg m^−3^; here we assume^32,33^ ρ_*PMMA*_ = 1190; ρ_*PET*_ = 1400; ρ_*PC*_ = 1200; ρ_*Alu*_ = 2700; and ρ_*Steel*_ = 7900).

## Results and discussion

### Growth rates as a function of pressure, pCO_2_ and pN_2_

**Fig. 2:**
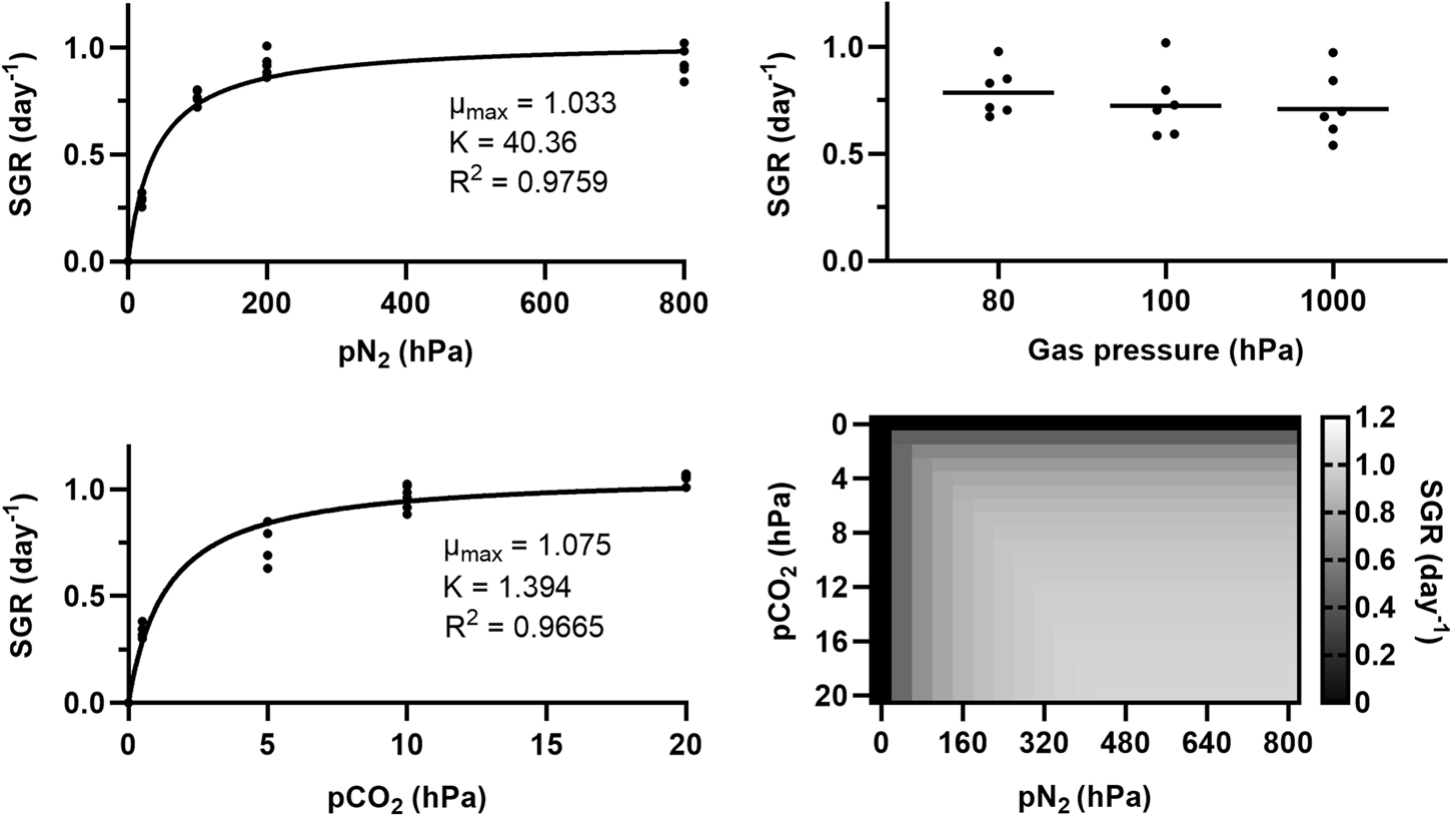
Impact of the partial pressures of CO_2_ and N_2_, and of the total pressure, on the growth rates of *Anabaena* sp. An equilibrium is assumed between the atmosphere and the culture medium. Left: Specific growth rate (SGR) as a function of pN_2_ under 10 hPa of pCO_2_ (top), or as a function of pCO_2_ under 800 hPa of pN_2_ (bottom). Dots correspond to experimental data (six biological replicates per condition); lines represent equations, analogous to Monod’s, whose parameter values are indicated. Top right: SGR as a function of total pressure, in an Ar atmosphere (carbon and nitrogen sources were provided in the culture medium). Horizontal lines show average values for six biological replicates (dots); differences are not significant (two-way ANOVA, *p* > 0.05). Bottom right: Heatmap showing SGR as a function of pCO_2_ and pN_2_.

The specific growth rates of *Anabaena* sp. as a function of pCO_2_ (under 80% N_2_) and pN_2_ (under 1% CO_2_), under one bar of total pressure, were determined after cultivating cyanobacteria in Atmos under controlled atmospheric conditions. Results are shown in Fig. 2, left. As cultivation under 5% CO_2_ resulted in specific growth rates (0.89 ± 0.06 day^−1^) lower than under 2% CO_2_, the associated data was excluded from the analysis, whose scope did not include above-optimal partial pressures. For either gas, the dependency of growth rates on partial pressure can be described with an equation analogous to Monod’s (assuming that the culture medium and gas phase are in equilibrium):

21 $${{{\mu }}}_{i}={{{\mu }}}_{{max} (i)}\,\cdot\,\frac{{p}_{i}}{{p}_{i}+{K}_{i}}$$

In Eq. (21), *i* refers to either CO_2_ or N_2_, µ_*i*_ (day^−1^) is the gas *i*-dependent specific growth rate, ${\mu }_{\max (i)}$ (day^−1^) the maximum growth rate inferred from experiments pertaining to gas *i* (hence the two slightly differing values), ${p}_{i}$ (hPa) the partial pressure of gas *i*, and *K*_*i*_ (hPa) – which is analogous to a half-velocity constant – the partial pressure of gas *i* for which ${\mu }_{i}=0.5\cdot{\mu }_{\max (i)}$. Values for ${\mu }_{\max (i)}$ and *K*_*i*_ are shown in Fig. 2, left. As the experiments we performed were short-term (four days), one cannot exclude that longer-term exposure to given atmospheric conditions could have effects (e.g., through physiological adaptation) that draw growth rates away from our predictions.

The effects of total atmospheric pressure itself (independently of pCO_2_ and pN_2_) on the specific growth rates of *Anabaena* sp. were assessed by cultivating cyanobacteria under 80, 100 or 1000 hPa. The lower end of the tested pressure range was constrained by a need to avoid excessive evaporation, or even cavitation, at 30 °C. While we considered relying on atmospheric carbon and nitrogen, and adjusting their concentrations to maintain their partial pressures constant across the tested pressure range, we rather relied on an Ar-only atmosphere (and on providing carbon and nitrogen as carbonates and nitrates in the liquid medium) for two main reasons: first, because water vapour was not controlled and, at low total pressures, would have largely impacted the concentration of other gases in the vessels; second, because changes in total pressure may have had an effect on the use of gaseous carbon and nitrogen even at constant partial pressures – through, for instance, changes in gas diffusion and solubility^12^. Specific growth rates (Fig. 2, top right) were not significantly affected by total pressure.

Specific growth rates in given atmospheric conditions can therefore be predicted based on pN_2_ and pCO_2_. Such predictions are shown as a heatmap in Fig. 2, bottom right. For each square, the specific growth rate is calculated as the minimum between that predicted based on pCO_2_ (assuming non-limiting N_2_; see Fig. 2, bottom left) and that predicted based on pN_2_ (assuming non-limiting CO_2_; then, µ_*maxN2*_ = 1.131 and *K*_*N2*_ = 51.99). One can note that above 400 hPa of pN_2_ and 10 hPa of pCO_2_, hardly any increase in growth rates can be obtained by increasing the partial pressure of either gas.

### Pressure required to support given growth rates

**Fig. 3:**
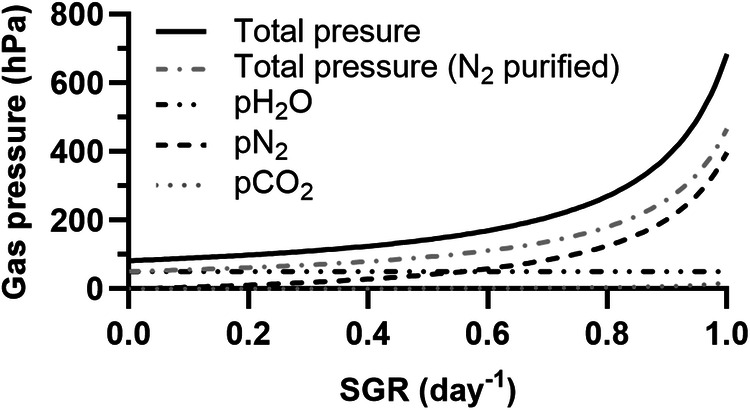
Minimum pCO_2_, pN_2_, partial pressure of water vapour (pH_2_O) and total gas pressure required to support the photoautotrophic, diazotrophic growth of *Anabaena* sp. at given specific growth rates (SGR). An equilibrium is assumed between the atmosphere and the culture medium. Two scenarios are considered for the total pressure: one where N_2_ is provided as Martian atmospheric gases purified from CO_2_ (total pressure), the other where N_2_ has been further purified by removing Ar and trace gases (total pressure [N_2_ purified]).

If cyanobacteria are cultivated using resources naturally available on Mars, factors unrelated to atmospheric conditions are likely to limit growth. These may, for instance, be the presence of toxic compounds (e.g., perchlorates) in the liquid phase and the limited availability of elements leached from regolith^4^. For the sake of resource efficiency, N_2_ and CO_2_ could then be provided at the lowest partial pressures which can sustain the growth rates supported by the other factors. These partial pressures are given in Fig. 3. Other gases will contribute to the total pressure. One is water, whose vapour we assume to be saturating. Others may be Ar and gases found as traces in the Martian atmosphere, which would be sparged into the photobioreactor together with N_2_: while technologies exist that could be used to purify N_2_ beyond the removal of CO_2_, here we assume that the resources (notably the energy) required for it would exceed the resources saved by the associated decrease in pressure. The partial pressure of water vapour, as well as the minimum total gas pressure (with or without N_2_ purification), are shown in Fig. 3 as a function of specific growth rates.

One important limitation to these results is their reliance on the assumption that CO_2_ has been removed entirely from the source of N_2_ (e.g., with adsorbents and/or CO_2_ freezers), or reduced to the minimum levels required for the targeted growth rates. Pressurized, N_2_-enriched air may indeed be a byproduct of CO_2_ purification or conversion processes (envisioned for, e.g., propellant, polymer or O_2_ production)^36,37^. With technologies which are – or have been – under consideration^38–40^, enrichment would not lead to N_2_ concentrations as high as considered here. If the aim is to reach the values presented in Fig. 3, further purification should therefore be performed specifically for the needs of cyanobacterium cultivation. A solution which maximises resource efficiency may instead rely on excess pCO_2_ – to limit purification needs – and therefore higher total pressures. As the technologies to be deployed on Mars for atmosphere processing are in early development phases, and since gas needs unrelated to cyanobacterium cultivation are mission-dependent and as yet undetermined, this optimal solution currently cannot be defined and we assume a CO_2_-free source of N_2_.

### Impact of pressure requirements on photobioreactor mass

When assessing the extent to which the mass of a photobioreactor on Mars could be reduced by minimizing the inner gas pressure (without CO_2_ or N_2_ becoming limiting), we considered a simple system: a bubble column photobioreactor with a liquid phase of one cubic metre.

**Fig. 4:**
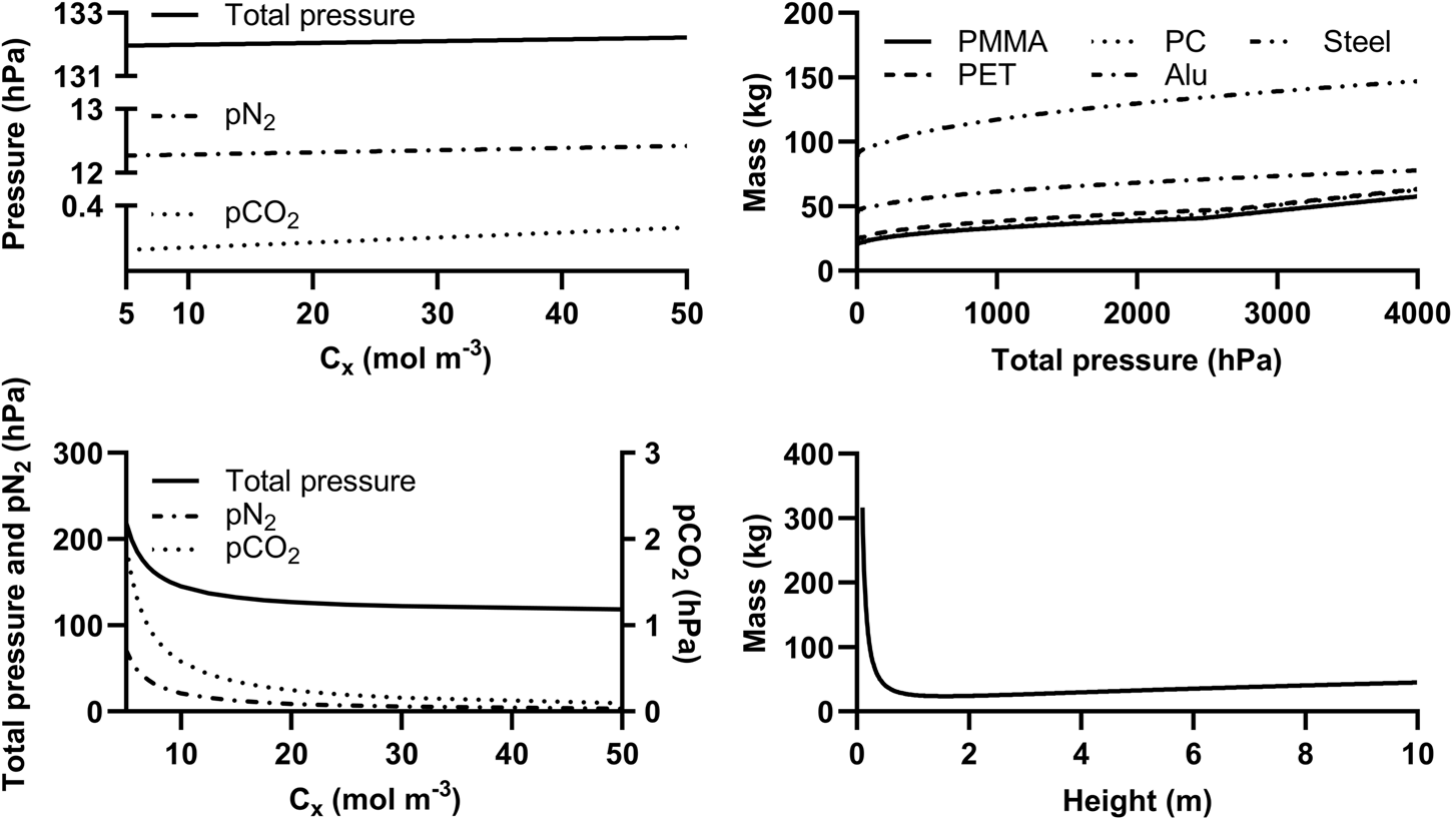
Impact of pressure requirements on the minimal mass of a photobioreactor’s outer walls. The assumed photobioreactor is a bubble column with a liquid phase twice as high as wide (except for the bottom-right panel) and of one cubic metre, a gas phase increasing the total height by one tenth, and a maximal gas superficial velocity of 0.08 m s^−1^. The ambient (outer) pressure is assumed to be 6 hPa. Top left: Minimal total pressure (including gas and hydrostatic pressures), pN_2_ and pCO_2_ required, as a function of biomass concentration (C_x_), to maintain a specific growth rate of 0.2 day^−1^. Bottom left: Minimal total pressure, pN_2_ and pCO_2_ required, as a function of C_x_, to maintain a biomass productivity of 100 g m^-3^ day^−1^ (assuming a molar mass of 32.965 g mol_x_^−1^ for *Anabaena* sp., as previously assessed^43^). Top right: Minimal mass of a photobioreactor’s outer walls required as a function of total pressure, for different materials. Bottom right: Minimal mass of a PMMA photobioreactor’s outer walls required to withstand an inner pressure of 100 hPa, as a function of reactor height (under a constant volume of 1 m^3^).

We calculated the total inner pressure required to maintain a given growth rate under a constant productivity, or a given productivity under a constant growth rate, in that reactor. This inner pressure has two main components: the hydrostatic pressure of the liquid medium and the gas pressure. The latter component can be calculated from the relationship given above between growth rates and atmospheric parameters. However, this relationship assumes that the gas and liquid phases are in equilibrium, and that consumed gases are replenished immediately. In practice, given that the superficial gas velocity is constrained (notably by the need to limit cell damage caused by shear stress), the required pCO_2_ and pN_2_ depend on the target productivity. We found the intensity of this dependence to be low (Fig. 4, top left): if productivity is to be increased by a factor of 10 by increasing the biomass concentration from 5 to 50 mol_x_ m^−3^ under a constant specific growth rate of 0.2 day^−1^ (a value more realistic, when cyanobacteria rely on regolith, than the maximum obtained in the experiments presented here^4^), the total pressure need not increase by more than 2%. Conversely, the pressure required to maintain a given level of productivity is highly dependent on growth rates (and therefore on cell density). As shown in Fig. 4, bottom left, the total inner pressure required to maintain a productivity of 100 g m^−3^ day^−1^ is reduced by a factor of four when the biomass concentration is increased tenfold (from 5 to 50 mol_x_ m^−3^). One should note that factors independent of atmospheric conditions (e.g., light penetration and nutrient availability) constrain the achievable cell concentrations and growth rates. This is outside the scope of the present work.

Past a (material-dependent) pressure threshold, which is due to a minimum material thickness required for manufacturing and handling, the minimal mass of the photobioreactor’s outer walls is a function of inner total pressure: thicker walls are required to withstand higher inside-outside pressure differences. Results for different materials are shown in Fig. 4, top right. These predictions are valid only for the assumed reactor design; Fig. 4, bottom right displays, as an example, the expected minimal mass of a PMMA photobioreactor’s outer walls, under constant pressure (100 hPa) and volume (1 m^3^), over a range of height values. There, mass is affected by changes in the volume-to-surface ratio. However, the involved orders of magnitude suggest that the reduction in the mass of a photobioreactor’s outer walls allowed by growing cyanobacteria under a low pressure on Mars would be negligible compared to the overall equivalent system mass^41,42^ (ESM) of cyanobacterium cultivation^43^, and *a fortiori* of the overall mission. Decreasing the cultivation pressure can, however, be expected to significantly improve the ESM by reducing the need to process gases from the Martian atmosphere.

### Reducing atmosphere processing requirements

Samples from the Martian atmosphere must be processed before they can be used as carbon and nitrogen sources: the total pressure and pN_2_, at least, must be increased. Technologies enabling such processes on Earth are well-proven^44^, and others are under development for gas pressurization and separation on Mars^36,39^. However, gas processing is resource intensive. If it is carried out entirely and exclusively for the purposes of cyanobacterium cultivation, relying on a gas phase in the photobioreactor which deviates no more than necessary from Mars’s atmosphere will be critical to the minimization of resource requirements. This may not be the case: other processes are expected to rely on atmospheric gases and their optimization would most likely be joint. If the exhaust gas from a CO_2_ purification system is used that contains 40% N_2_, for instance, a total gas pressure of circa 30 hPa would provide high enough a pN_2_ to support specific growth rates of 0.2 day^−1^.

A limit on total pressure which results in limiting pN_2_ may also be set without affecting the overall productivity. Indeed, the growth of cyanobacteria cultivated through ISRU is expected to be limited, from a given biomass concentration, by the availability of nutrients released from Martian regolith^43^. At this concentration, productivity would therefore be proportional to the nutrients’ release rates and growth rates be very low (and, for a given productivity, inversely proportional to biomass concentrations). While, early in a cultivation run – at low cell concentrations, when nutrients released by regolith in the medium have not yet been depleted –, growth rates could be high, limiting them by keeping pN_2_ and pCO_2_ (and therefore the total pressure) low would only delay the moment when growth is limited by nutrients leached by the regolith. It would not reduce the productivity averaged over the cultivation period.

Finally, if cyanobacteria are grown non-diazotrophically (e.g., by providing nitrogen recycled from organic waste), little to no processing would be required to provide a non-limiting pCO_2_ from the Martian atmosphere. Only a slight increase in the total pressure (to 80 hPa or less) would be required, to avoid partial pressure-independent inhibition by hypobaria, for which little more than a saturating water vapour might suffice.

To conclude: reducing the stress on a Martian photobioreactor’s outer walls by reducing pressure can allow for a reduction in hardware mass, but that reduction would likely be negligible next to the overall ESM of the cultivation system. However, and while providing quantitative estimates of the required resources would be highly tentative, minimizing the need for atmosphere processing may have a much larger impact. This minimization can be performed by processing gases no further than strictly required to support the targeted growth rates – the conditions required for which can be assessed based on the data presented here – and by targeting growth rates which maximize resource efficiency.

## Data Availability

Any further data that support the findings of this study will be made available by the authors upon reasonable request.
